# A Lasso and a Regression Tree Mixed-Effect Model with Random Effects for the Level, the Residual Variance, and the Autocorrelation

**DOI:** 10.1007/s11336-021-09787-w

**Published:** 2021-08-14

**Authors:** Steffen Nestler, Sarah Humberg

**Affiliations:** University of Münster, Institut für Psychologie, Fliednerstr. 21, 48149 Münster, Germany

**Keywords:** mixed-effect models, longitudinal data, within-person variability, lasso regression, regression trees

## Abstract

Research in psychology is experiencing a rapid increase in the availability of intensive longitudinal data. To use such data for predicting feelings, beliefs, and behavior, recent methodological work suggested combinations of the longitudinal mixed-effect model with Lasso regression or with regression trees. The present article adds to this literature by suggesting an extension of these models that—in addition to a random effect for the mean level—also includes a random effect for the within-subject variance and a random effect for the autocorrelation. After introducing the extended mixed-effect location scale (*E-MELS*), the extended mixed-effect location-scale Lasso model (*Lasso E-MELS*), and the extended mixed-effect location-scale tree model (*E-MELS trees*), we show how its parameters can be estimated using a marginal maximum likelihood approach. Using real and simulated example data, we illustrate how to use E-MELS, Lasso E-MELS, and E-MELS trees for building prediction models to forecast individuals’ daily nervousness. The article is accompanied by an R package (called mels) and functions that support users in the application of the suggested models.

The digitalization of almost all areas of human life has led to the availability of a wide range of longitudinal data for many individuals (see Harlow & Oswald, 2016). In order to test psychological hypotheses or to forecast the behavior or the emotions of individuals with intensive longitudinal data, standard machine-learning methods such as regularized regression models (Hastie et al., 2009; McNeish, 2015), support vector machines (Schölkopf & Smola, 2002), boosting (James et al., 2013), random forests (Kuhn & Johnson, 2013; Strobel et al., 2009), and neural networks (Goodfellow et al., 2016) are often used. However, a problem with most of these machine-learning approaches is that they were developed for cross-sectional data and not for longitudinal data that are nested in individuals.

In recent years, extensions of these classical methods that consider the hierarchical structure of longitudinal data have thereby been proposed. These extensions are based on longitudinal mixed-effect models that have been, for example, combined with Lasso regression (e.g., Groll & Tutz, 2014; Li et al., 2018; Pan & Huang, 2014; Schelldorfer et al., 2011) or regression trees (e.g., Hajjem et al., 2011; Sela & Simonoff, 2012; Stegmann et al., 2018). In contrast to the classical machine-learning methods, these extensions allow for between-person differences in mean levels and therefore have a higher predictive power (see, e.g., the simulation in Sela & Simonoff, 2012). However, psychological research shows that individuals differ in how much they fluctuate around their personal mean (within-person variability, see, e.g., Baird et al., 2006; 2017; Geukes et al., 2017) and in the extent to which the actual value of the person is predicted by the person’s previous value (autocorrelation, e.g., Jahng et al., 2008; Wang et al., 2012), these extensions therefore also share a crucial limitation, because they do not account for such between-person differences in the Level 1 residual variance and the autocorrelation. There are extensions of the *mixed-effect* model that can be applied to examine interindividual differences in the levels, within-person variability, and autocorrelation (e.g., Gasimova et al., 2014; Hamaker et al., 2018; Hedeker et al., 2008; Nestler, 2020), but these models have not yet been combined with Lasso regression or regression trees. This is surprising because, by additionally considering the between-person differences in the Level 1 residual variance and in the autocorrelation, the predictive performance of the *Lasso mixed-effect model* or the *tree longitudinal mixed-effect model* might be improved. For example, allowing for such between-person differences should lead to more accurate forecasts (in terms of mean squared error, for example) when we use the model to predict the outcome value on a new day for a person that was used to estimate a model’s parameters (i.e., forecasting within a person across time). Furthermore, the forecast might also be more accurate for persons that were not used for parameter estimation (i.e., forecasting across persons).

The aim of our article is therefore to introduce combinations of (a) Lasso regression and (b) regression trees with an extension of the longitudinal mixed-effect model that—in addition to varying in the intercept (individuals differ in their “level” of the outcome variable)—allows individuals to differ in the within-person variance and the autocorrelation. To this end, we first describe the illustrative example that we use throughout the article. We continue with a description of the longitudinal mixed-effect model, and then explain the factors that affect the forecasting behavior of this model. Thereafter, we explain why the aforementioned extensions are better suited for tackling the different forecasting problems that can occur with longitudinal data. We then present the two combinations, and show how the two models’ parameters can be estimated with a maximum likelihood (ML) approach. Finally, we illustrate all models using a real and a simulated data example.

## Illustrative example

Throughout the following section, we use data from a study called FLIP (i.e., Fluctuations In Personality, Hofmann & Nestler, 2019) to illustrate the different models. FLIP is a daily diary study for which about 100 individuals were asked to fill out state measures of personality, affective states, and motivational states. Prior to and after the state assessments, they also answered questionnaires about different personality traits, took intelligence tests, provided demographic information, etc. In the following illustration and explanations, we focus on participants’ daily assessments of the item “I was nervous” (rated on a scale ranging from 1 = *not at all* to 6 = *very*), which was used to measure individuals’ nervousness.

The data from the nervousness item can be employed to examine a number of different research questions. For instance, we could investigate whether subjects’ average nervousness ratings differ across persons and whether these between-person differences in the mean level are related to certain personality traits such as neuroticism or extraversion (Baird et al., 2017; Geukes et al., 2017). Here, we take a more predictive stance and use the data to design a statistical model that can be employed to forecast a person’s nervousness on a new day. If we were dealing with cross-sectional data, we could use a number of different machine-learning methods for this, such as linear regression, regularized regression models (Hastie et al., 2009; McNeish, 2015), support vector machines (Schölkopf & Smola, 2002), boosting (James et al., 2013), random forests (Kuhn & Johnson, 2013; Strobel et al., 2009), and neural networks (Goodfellow et al., 2016). However, these models are not suitable for longitudinal data, because they assume that the data is independently and identically distributed and this assumption is violated when time points are nested within individuals. Instead, a more appropriate approach is to use extensions of the mixed-effect model for longitudinal data.

## Mixed-effect models for longitudinal data

In the case of longitudinal data, the predictors^1^ can be variables that are *constant* across time (e.g., a person’s gender), or variables that *vary* with time. The time-varying variables can comprise contemporaneous variables (e.g., a person’s anxiety value at time point *t*) and/or lagged variables (e.g., the anxiety value at time point $t - 1$). One can also include time-varying variables that code time (e.g., a linear time variable) or seasonal variables (e.g., weekday). We use the $T = T_1 + \cdots + T_I$ data points of the *I* individuals to determine the prediction model and therefore refer to these data as the *training sample*.

The general mixed-effect model for the $T_i$ repeated observations $\varvec{y}_i$ of person *i* (called *longitudinal mixed-effect model* throughout this article) is given by:

1 $$\begin{aligned} \varvec{y}_i = \varvec{X}_i\varvec{\beta } + \varvec{Z}_i\varvec{b}_i + \varvec{\epsilon }_i. \end{aligned}$$

In our example, $\varvec{y}_i$ contains all nervousness values of person *i*. $\varvec{X_i}$ is a $T_i$ x $(p + 1)$ vector of predictor values of person *i* (including a column of 1s for the intercept) and $\varvec{\beta }$ is a $(p+1)$ x 1 vector containing regression weights. The entries of $\varvec{\beta }$ are also called *fixed effects*. $\varvec{Z_i}$ is a $T_i$ x *k* design matrix for the random effects that are contained in the *k* x 1 vector $\varvec{b}_i$. We assume that $\varvec{b}_i$ follows a multivariate normal distribution with an expectation of zero and a *k* x *k* covariance matrix $\varvec{\Phi }$. Finally, $\varvec{\epsilon }_i$ is a $T_i$ x 1 vector of residual terms that is also assumed to be normally distributed with expectation of zero and covariance matrix $\varvec{\Sigma }$ of size $T_i$ x $T_i$.

A special case of the longitudinal mixed-effect model that is often used is the random-intercept model:

2 $$\begin{aligned} \varvec{y}_{i} = \varvec{X}_i\varvec{\beta } + \varvec{1}_{T_{i}} \tau _{i} + \varvec{\epsilon }_i . \end{aligned}$$

It is a special case of Eq. (1) where $\varvec{Z_i}$ is a column vector of 1s (hence *k* = 1), $\varvec{b}_i$ is a single number, $\tau _i$, and $\varvec{\Phi }$ contains only one element, $\phi ^2_{\tau }$. In this case, $\tau _i$ is person *i*’s deviation from the intercept across the sample. When no predictors are included in the model, the intercept reflects the mean of all outcome values across the persons in the training sample, and $\tau _i$ is the deviation of person *i* from this average *y* value, also called a person’s level. $\phi ^2_{\tau }$ is a measure of the extent to which the persons differ in $\tau _i$. Furthermore, because intensive longitudinal data is collected a few minutes, hours, or days apart, it is reasonable to assume that the error terms are autocorrelated ( cf. Verbeke & Molenberghs, 2009). Whereas different types of autocorrelated error structures are possible (see Hedeker & Gibbons, 2006, Chapter 7, for an overview), for the purposes of demonstration in this paper, we focus on a lag-1 autoregressive error process that models the covariance matrix of $\varvec{\epsilon }_i$ as

3 $$\begin{aligned} \varvec{\Sigma } = \frac{\sigma _{\epsilon }^{2}}{1 - \rho ^2} \begin{pmatrix} 1 &{} \rho &{} \rho ^{2} &{} ... &{} \rho ^{T_i} \\ \rho &{} 1 &{} \rho &{} ... &{} \rho ^{T_i - 1}\\ \rho ^2 &{} \rho &{} 1 &{} ... &{} ... \\ ... &{} ... &{} ... &{} ... &{} \rho \\ \rho ^{T_i} &{} ... &{} ... &{} \rho &{} 1\\ \end{pmatrix} . \end{aligned}$$

where $\sigma _{\epsilon }^{2}$ is the variance of the residual terms, and $\rho$ is the autocorrelation. For both of these parameters, the values are estimated to be the same for all individuals.

### Predicting and Forecasting with Longitudinal Mixed-Effect Models

Having obtained the mixed-effect model estimates, we can use them to predict a person’s outcome value. Following the mixed-effect prediction literature (see Afshartous & de Leeuw, 2005; Chi & Reinsel, 1989; Sela & Simonoff, 2012; Skrondal & Rabe-Hesketh, 2009), we differentiate between three types of prediction tasks: First, we could use the model to predict the outcome of a person who was part of the training sample for *H* time steps in the future (*Task 1*). In our example, we could forecast the nervousness of a person in the training sample on subsequent days. When $H = 1$, the value at the next time step is predicted. This is called a one-step forecast. Cases in which $H > 1$ are referred to as multiple-step forecasts (Hyndman & Athanasopoulos, 2018, Chapter 3). Task 1 predictions can be computed with^2^ (see Chi & Reinsel, 1989):

4 $$\begin{aligned} \hat{y}_{i, T_i + H} = \varvec{X}_{i, T_i + H} \varvec{\hat{\beta }} + \varvec{Z}_{i, T_i + H}\hat{\varvec{b}}_i + \hat{\rho }^{H}\hat{\varvec{\epsilon }}_{i,T_i} \end{aligned}$$

where we assume that $\varvec{X}_{i, T_i + H}$ and $\varvec{Z}_{i, T_i + H}$ are known, $\varvec{\hat{\beta }}$ and $\hat{\rho }$ are the fixed effects and the autocorrelation, respectively, estimated with the training data. $\hat{\varvec{b}}_i$ is an estimate of person *i*’s random effect terms obtained with the training data and $\hat{\varvec{\epsilon }}_{i,T_i}$ is an estimate of person *i*’s Level 1 residual at the $T_i$th time point.

Second, we could use the model to predict the values of a completely new person for whom no past observations are available (*Task 2*). As an example, we could use the model to predict the nervousness of a person who was not in the training sample and for whom no past observations are available. In this case, no random effect estimate and no estimate of the Level 1 residual are available, so that the *H*-step forecast is just $\hat{y}_{i,H} = \varvec{X}_{i,H} \varvec{\hat{\beta }}$. Finally, the model could also be used to predict the values of a new person who was not part of the training sample, but for whom past observations are available (*Task 3*). For example, we could predict the nervousness on a subsequent day for a person that we did not use to determine the prediction model but for whom past observations of nervousness are available. In this case, we would estimate the person’s random effect $\hat{\varvec{b}}_i$ and the residual $\hat{\varvec{\epsilon }}_{i,T_i}$ using the mixed-effect model estimates obtained with the training sample. Then, we use Equation (4)—analogously to Task 1—to obtain a forecast for the new person on a new day.

Note that Task 1 is the typical focus of prediction in the forecasting literature, because it explicitly considers temporal information for the trained persons (e.g., the previous values of a person or the autocorrelation, see Hyndman & Athanasopoulos, 2018). The other two prediction tasks are typically not considered in the “conventional” forecasting literature because they do not involve temporal information, as in Task 2, or because they are based on a very strict stationarity assumption, as in Task 3. However, Tasks 2 and 3 are among the subjects of investigation in the multilevel prediction literature, and we think that they are relevant for practical applications in which nothing is known about individuals except that they belong to the same population as the training sample (see Jiang, 2007). We therefore cover all three prediction tasks in this article and examine how our model extensions can be used to approach these tasks. We will use the term *H*-step forecasts to refer to predictions within Tasks 1, 2, and 3 (where $T_i = 0$ in Task 2, because no previous information about the person is available).

Equation (4) shows that it is important to take the nested longitudinal data structure into account when one is interested in accurate predictions for Task 1 and 3. For instance, in the case of a random intercept model, a prediction that takes individual differences in the person’s intercept $\hat{\varvec{b}}_i$ into account (i.e., that uses information about whether a *specific* person has systematically higher or lower values in *y* than others) should be better than a prediction based on the expected value across all individuals. Thus, when level information is available, as in Task 1 and 3, it should be considered in the prediction model, but this is not done in standard machine-learning models. Furthermore, failing to consider the autocorrelation of the outcome values affects the quality of the forecast, and this effect is larger, the closer the time point to be predicted is to the available data about the person.

One limitation to using the longitudinal mixed-effect model for prediction is—similar to a standard regression model—that it assumes the data can be well-described by a linear function of the fixed effects and random effects. Hence, if the true data-generating process takes a nonlinear form, future values will be poorly predicted; this applies to predictions for all three types of tasks. Another problem relevant for all three prediction tasks is that $\varvec{X}_i$ can contain many predictors, such as contemporaneous and lagged variables, variables coding time, seasonal variables (e.g., weekdays), or person-level variables. Hence, one has to select the best predictors to use in the model (cf., Hyndman & Athanasopoulos, 2018, Chapter 5). This also reduces the probability of overfitting, which would occur if the model predicts the outcome values in the training sample almost perfectly but only poorly predicts new values (see McNeish, 2015, for an introduction). This occurs because the model partly captures irrelevant random deviations in the training data (i.e., noise), with the consequence that the model does not generalize to new data. The next two sections discuss how these problems have been addressed in the literature on mixed-effect modeling.

### A Lasso Mixed-Effect Model

As a solution to the overfitting problem, several researchers have suggested that longitudinal mixed-effect models be combined with Lasso regression (e.g., Fan & Li, 2012; Li et al., 2018; Schelldorfer et al., 2011; Pan & Huang, 2014; Groll & Tutz, 2014; Schelldorfer et al., 2014). Similar to the standard Lasso model (Hastie et al., 2009; McNeish, 2015), the basic idea is to apply a penalty function *P* to the fixed-effect coefficients during estimation that shrinks small coefficients to exactly zero. This is achieved by setting a constraint on the sum of the absolute values of the coefficients $P(\varvec{\beta }) = \uplambda \sum _{j = 1}^{p} |\beta _{j}|$, where $\uplambda \ge 0$ is the regularization parameter. This penalty term for the fixed effects is subtracted from the log-likelihood function of the longitudinal mixed-effect model, that is then used to obtain the maximum likelihood parameters (see Hedeker & Gibbons, 2006, for the matrix formula of the log-likelihood function of the mixed-effect model):

5 $$\begin{aligned} \text {logL}_{\uplambda ,\text {MEM}} = \text {logL}_{\text {MEM}} - \uplambda \sum _{j = 1}^{p} |\beta _{j}| . \end{aligned}$$

The regularization parameter $\uplambda$ controls the amount of shrinkage. When $\uplambda$ is zero, the Lasso coefficients are identical to the fixed effects obtained in a standard longitudinal mixed-effect model. By contrast, the higher $\uplambda$ is, the larger is the number of fixed effects that are set to zero. Thus, Lasso results in a “sparse” fixed-effect vector $\varvec{\beta }$ where some, or even the majority of coefficients, are zero, depending on the choice of $\uplambda$. It is this removal of negligible predictors that helps to avoid overfitting.

### A Mixed-Effect Model Tree

**Fig. 1:**
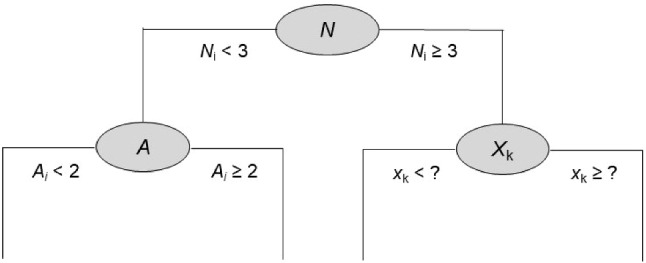
A simple tree (see text for explanations). N = neuroticism, A = Agreeableness.

To allow that the data can be modeled as a nonlinear function of the fixed effects and random effects, researchers have suggested using a combination of a cross-sectional regression tree and the longitudinal mixed-effect model (see, e.g., Fu & Simonoff, 2015; Hajjem et al., 2011; Sela & Simonoff, 2012; Stegmann et al., 2018). A *cross-sectional* regression tree for a predictor matrix $\varvec{X}$ and outcome variable $\varvec{y}$ (see Strobel et al., 2009; James et al., 2013, for introductions) divides the space spanned by the predictors in $\varvec{X}$ into *G* subsets or regions. To build a tree, one first needs to define how the predicted value $\hat{y}$ of a person should be computed. Typically, a person’s predicted value is the mean of the outcome values of the training sample individuals who fall into a specific region *G* defined by the constellation of predictor values. Then an iterative process of region building begins (called recursive partitioning) in which the first step is to select a single predictor variable $X_j$ from $\varvec{X}$ and a value $s_j$ that split the space spanned by $X_j$ into two regions ($X_j < s_j$ and $X_j \ge s_j$). The variable $X_j$ and the value $s_j$ are chosen such that in comparison with all other predictors in $\varvec{X}$ and split values, it is accompanied by the smallest prediction error (i.e., the sum of the squared differences between the actual and predicted value). In our example (see Figure 1), if dividing the space into the two neuroticism regions (“individuals with N < 3” and “individuals with N $\ge$ 3”) resulted in the lowest prediction error, neuroticism together with the value 3 would be selected as the first splitting variable. In the second step, the next predictor $X_{k}$ with a split value $s_k$ is selected in order to split the data further (e.g., agreeableness and the value 2). This second step of splitting is done *within* the two regions that were identified for the predictor selected in the first step. In our example, splitting with respect to agreeableness is thus done within each of the two regions of neuroticism. This process is repeated until a minimum number of persons (e.g., 20 persons) in each leaf of the tree is reached (other stopping criteria are possible). A trained tree tends to over-fit the training data. Therefore, one often deletes nodes from the fully grown tree and the resulting sub-tree is then used for prediction (this is called pruning, see Hastie et al., 2009, Chapter 9).

The final regression tree consists of *G* regions $R_1$, $R_2$, ..., $R_G$. The predicted value of a person is the mean of the $\varvec{y}$-values of the individuals in the training sample that fall into $R_g$ because of their predictor values contained in $\varvec{x}_i$. As a consequence, the final tree can be displayed as a regression model with *G* dummy variables:

6 $$\begin{aligned} y_i = \sum _{g = 1}^{G} 1_{R_{g}}(\varvec{x}_i) \varvec{\mu }_g + \epsilon _i \end{aligned}$$

where $1_{R_{g}}$ denotes the indicator function that takes a value of 1 when the argument $\varvec{x}_i$ lies in region $R_{g}$ and 0 otherwise. This is equivalent to saying that person *i* falls into $R_g$ because of her or his predictor values. $\varvec{\mu }_g$ is the mean of the *y* values in region $R_g$.

Now, when the aim is to estimate a *longitudinal mixed-effect model tree*, the algorithm iterates between two steps: First, assuming that the random effects, $\hat{\varvec{b}}_i$, of the individuals are known, one estimates a cross-sectional regression tree with the residuals that were freed from the random effects (i.e., these are not the Level 1 residuals)

7 $$\begin{aligned} \varvec{\hat{\epsilon }}_i = \varvec{y}_i - \varvec{Z}_i \varvec{\hat{b}}_i . \end{aligned}$$

This is plausible because the residuals do not contain any information about between-person differences with regard to these random effects. Second, the tree from the previous step is incorporated into the multilevel growth model using dummy variable regression model:

8 $$\begin{aligned} \varvec{y}_i = y_i = \sum _{g = 1}^{G} 1_{R_{g}}(\varvec{x}_i) \varvec{\mu }_g + \varvec{Z}_i\varvec{b}_i + \varvec{\epsilon }_i \end{aligned}$$

and the random effects and variance terms of the growth model are estimated using standard multilevel software. The algorithm (called RE-EM tree, see Sela & Simonoff, 2012, but also Hajjem et al., 2011) alternates between these two steps until it converges. As for cross-sectional data, the result is a tree that divides the space spanned by the time-constant and time-varying predictors into regions. Also, the average of the outcome values in a region is taken as the predicted outcome value for new individuals. The only difference is that observations from different time points for the same person can fall into different regions.

### Between-Person Differences in the Level 1 Variance and the Autocorrelation

Simulation studies show that the Lasso mixed-effect model and the mixed-effect model tree provide better predictions than the standard cross-sectional approaches ignoring the hierarchical data structure and than the longitudinal mixed-effect model (see, e.g., Sela & Simonoff, 2012). However, we believe that the predictive performance of the models can be further improved by taking into account *interindividual differences* in the Level 1 residual variance, $\sigma _{\epsilon }^{2}$, and in the autocorrelation, $\rho$. Research shows that such differences exist and that they are systematically associated with other variables (e.g., Baird et al., 2017; Geukes et al., 2017; 2017; Jahng et al., 2008). Hence, considering them in the model should improve the accuracy and the precision of the predictions. With regard to accuracy, Equation (4) clearly shows that considering a person-specific autocorrelation should yield more precise forecasts. Furthermore, a person-specific autocorrelation, but also a person-specific residual variance may affect forecast accuracy more indirectly, because simulation results show that omitting both types of interindividual differences from the mixed-effect model specification can lead to biases in the estimates of $\varvec{\Phi }$ (e.g., the intercept variance $\phi _\tau ^2$; Leckie et al., 2014; Schuurman et al., 2016). This in turn should bias the random effect estimates $\hat{\varvec{b}}_i$, because $\varvec{\Phi }$ is contained in the formula to compute them. An estimate of $\varvec{b}_i$ is given by

9 $$\begin{aligned} \hat{\varvec{b}}_i = \varvec{\Phi }\varvec{Z}_{i}'\varvec{V}^{-1}(\varvec{y}_i - \varvec{X}_i\hat{\varvec{\beta }}) \end{aligned}$$

where $\varvec{V}$ is the covariance matrix of $\varvec{y}$. However, Equation (4) shows that $\hat{\varvec{b}}_i$ is important for calculating the forecast, which implies that a more accurate estimate $\hat{\varvec{b}}_i$ (i.e., the model is correctly specified) should increase the precision of the forecast.

With regard to precision, interindividual differences in the Level 1 variance and autocorrelation should also influence the forecast error variance. When we assume that the variance components of the model are known, the variance of the forecast error in case of prediction Tasks 1 and 3 is (Frees, 2004, Chapter 4; Skrondal & Rabe-Hesketh, 2009):

10 $$\begin{aligned} \text {var}( \hat{y}_{i, T_i + H} - y_{i, T_i + H} )&= \left( \varvec{X}'_{i, T_i + H} - \varvec{C}\varvec{V}^{-1}\varvec{X}_{i} \right) \text {cov}({\hat{\varvec{\beta }}}) \left( \varvec{X}'_{i, T_i + H} - \varvec{C}\varvec{V}^{-1}\varvec{X}_{i} \right) ' \nonumber \\&+ \varvec{Z}'_{i, T_i + H} \left( \varvec{\Phi } - \varvec{\Phi }\varvec{Z}'_{i}\varvec{V}^{-1}\varvec{Z}_{i}\varvec{\Phi } \right) \varvec{Z}_{i, T_i + H} + \text {var}(\epsilon _{i, T_i + H}) \end{aligned}$$

where $\text {cov}({\hat{\varvec{\beta }}})$ is the covariance matrix of the fixed-effect estimates and

11 $$\begin{aligned} \varvec{C}&= \varvec{Z}'_{i, T_i + H}\varvec{\Phi }\varvec{Z}'_{i} + \text {cov}(\epsilon _{i, T_i + H},\epsilon _{i}) \end{aligned}$$

where $\text {cov}(\epsilon _{i, T_i + H},\epsilon _{i}) = \sigma ^2( \rho ^{T_i + H - 1}, \rho ^{T_i + H - 2}, \cdots , \rho ^{H})$ (and $\sigma ^{2} = \sigma ^{2}_{\epsilon }/(1-\rho ^2)$). Consequently, considering a person-specific residual variance or person-specific autocorrelation should yield a smaller prediction error variance (i.e., the first term in Equation (10) should be smaller) than if all persons’ predictions were based on the same value for the residual variance and/or autocorrelation. A similar argument can be made for Task 2 predictions, because the variance for the prediction errors is (Skrondal & Rabe-Hesketh, 2009)

12 $$\begin{aligned} \text {var}( \hat{y}_{i, T_i + H} - y_{i, T_i + H} )&= \varvec{X}'_{i, T_i + H}\text {cov}({\hat{\varvec{\beta }}})\varvec{X}_{i, T_i + H} + \varvec{Z}'_{i, T_i + H}\varvec{\Phi }\varvec{Z}_{i, T_i + H} + \text {var}(\epsilon _{i, T_i + H}). \end{aligned}$$

Hence, when failing to consider interindividual differences in the Level 1 variance and the autocorrelation the estimate of the variance of their prediction errors should be less precise. The results of a small simulation study support this conjecture (see “Appendix A”).

## Extending the Longitudinal Mixed-Effect Model to Consider Between-Person Differences in the Level 1 Variance and the Autocorrelation

In this section, we present an extended longitudinal mixed-effect model that incorporates between-person differences in the residual Level 1 variance and the autocorrelation. To the best of our knowledge, this combination has not yet been proposed in the literature. However, it includes as a special case the mixed-effect location-scale model (MELS) that was suggested by Hedeker and colleagues (e.g., Hedeker et al., 2008; Hedeker et al., 2009; 2009) and that allows to consider between-person differences in the residual Level 1 variance, but not in the autocorrelation. It can also be considered a special case of the recently introduced dynamic structural equation model (Asparouhov et al., 2018; Hamaker et al., 2018). Due to this background, we will call our extension the extended mixed-effect location-scale model (E-MELS). After the description of the E-MELS, we will show how it can be combined with Lasso regression and regression trees.

### The Basic E-MELS

The E-MELS extends the longitudinal mixed-effect model (see Eq. 1) in that it allows the residual variance and the autocorrelation to differ between individuals. Specifically, we assume that the covariance matrix of the residual terms is person-specific:

13 $$\begin{aligned} \varvec{\Sigma }_{i} = \frac{\sigma _{\epsilon _{i}}^{2}}{1 - \rho ^2_{i}} \begin{pmatrix} 1 &{} \rho _{i} &{} \rho _{i}^{2} &{} ... &{} \rho _{i}^{T_i} \\ \rho _{i} &{} 1 &{} \rho _{i} &{} ... &{} \rho _{i}^{T_i - 1}\\ \rho _{i}^2 &{} \rho _{i} &{} 1 &{} ... &{} ... \\ ... &{} ... &{} ... &{} ... &{} \rho _{i}\\ \rho _{i}^{T_i} &{} ... &{} ... &{} \rho _{i} &{} 1\\ \end{pmatrix} , \end{aligned}$$

where $\sigma _{\epsilon _{i}}^{2}$ is person *i*’s residual variance, and $\rho _{i}$ is *i*’s autocorrelation. Both terms are defined as

14 $$\begin{aligned} \sigma _{\epsilon _{i}}^{2} = \text {exp} (s_{0} + \omega _{i}) \nonumber \\ \rho _{_{i}} = \text {tanh} (r_{0} + \iota _{i}) . \end{aligned}$$

Here, $s_{0}$ denotes the average of the logarithm of the Level 1 variance, and $r_{0}$ is the average of the inverse hyperbolic tangent of the autocorrelation across individuals. $\omega _{i}$ and $\iota _{i}$ are person-specific random effects, reflecting the extent to which person *i*’s residual variance and autocorrelation deviate from $s_{0}$ and $r_{0}$, respectively. We use the exp function to ensure that the residual variance remains positive. Similarly, we use the tanh (i.e., hyperbolic tangent) function to ensure that the autocorrelation remains in the interval from $-1$ to 1. Both functions are the default choices in the literature to keep the model parameters within their defined value ranges (see, e.g., Goodfellow et al., 2016; Hedeker et al., 2008).

In the following, we assume that $\varvec{v}_{i} = (\varvec{b}_{i}, \omega _{i}, \iota _{i})$ contains the random effects of a person *i* and that these terms are normally distributed with expectation zero and covariance matrix $\varvec{\Phi }$:

15 $$\begin{aligned} \varvec{\Phi } = \begin{bmatrix} \begin{array}{c|c} \varvec{\Phi }_{\varvec{b}} &{} \varvec{\Phi }_{\varvec{b},(\omega ,\iota )} \\ \hline \varvec{\Phi }_{\varvec{b},(\omega ,\iota )}' &{} \varvec{\Phi }_{(\omega ,\iota )} \\ \end{array} \end{bmatrix} . \end{aligned}$$

Here, $\varvec{\Phi }_{\varvec{b}}$ is the *k* x *k* covariance matrix of $\varvec{b}_i$ (i.e., the random effects of the mean structure), $\varvec{\Phi }_{\varvec{b},(\omega ,\iota )}$ contains the covariance terms between the random effects in $\varvec{b}$ and the random effect of the residual variance $\omega$ or the autocorrelation $\iota$, respectively. Finally, $\varvec{\Phi }_{(\omega ,\iota )}$ is a 2 x 2 matrix that contains the (co-)variance terms of $\omega _{i}$ and $\iota _{i}$, respectively. In case of a random-intercept model (Equation (2)), for example, $\varvec{\Phi }$ is

16 $$\begin{aligned} \varvec{\Phi } = \begin{bmatrix} \phi ^{2}_{\tau } &{} \phi _{\tau \omega } &{} \phi _{\tau \iota } \\ \phi _{\tau \omega } &{} \phi ^{2}_{\omega } &{} \phi _{\omega \iota } \\ \phi _{\tau \iota } &{} \phi _{\omega \iota } &{} \phi ^{2}_{\iota } \\ \end{bmatrix} . \end{aligned}$$

Here, $\phi ^{2}_{\tau }$ reflects the intercept variance, $\phi ^{2}_{\omega }$ indicates between-person differences in the residual variance, and $\phi ^{2}_{\iota }$ reflects the extent of the between-person differences in the autocorrelation. $\phi _{\tau \omega }$, $\phi _{\tau \iota }$, and $\phi _{\omega \iota }$ are the covariances between these random effects. For example, $\phi _{\tau \omega }$ reflects the relation between individuals’ mean levels and their residual variance.

Finally, we also assume that the distribution of the observed responses $\varvec{y}_i$ conditional on $\varvec{v}_i$ is normal with expectation $\varvec{\mu }_{\varvec{y}_i} = \varvec{X}_i \varvec{\beta } + \varvec{Z}_i\varvec{b}_{i}$ and covariance matrix $\varvec{\Sigma }_{i}$. This allows us to define the marginal likelihood of person *i*:

17 $$\begin{aligned} L_{i}(\varvec{\theta }) = f(\varvec{y}_i|\varvec{\theta }) = \int _{\varvec{v}_i} f( \varvec{y}_i| \varvec{\mu }_{\varvec{y}_i}, \varvec{\Sigma }_{i}, \varvec{v}_i ) f(\varvec{v}_i| \varvec{\Phi }) d \varvec{v}_i , \end{aligned}$$

and the marginal likelihood of the whole sample:

18 $$\begin{aligned} L(\varvec{\theta }) = \prod _{i = 1}^{I} L_{i}(\varvec{\theta }) \end{aligned}$$

where $\varvec{\theta }$ contains the parameters in $\varvec{\beta }$, $s_{0}$, $r_{0}$, and the parameters in $\varvec{\Phi }$. Finally, the marginal log-likelihood of the sample is

19 $$\begin{aligned} \text {logL}_{E-MELS}(\varvec{\theta }) = \sum _{i = 1}^{I} \text {logL}_{i}(\varvec{\theta }) = \sum _{i = 1}^{I} \text {log} \left[ \int _{\varvec{v}_i} f( \varvec{y}_i| \varvec{\mu }_{\varvec{y}_i}, \varvec{\Sigma }_{i}, \varvec{v}_i ) f(\varvec{v}_i| \varvec{\Phi }) d \varvec{v}_i \right] . \end{aligned}$$

The marginal log-likelihood is maximized to obtain the ML estimates of the parameters. No analytical solution exists for this maximization problem because one cannot analytically solve the integrals that appear in the log-likelihood function. Therefore, we suggest that researchers use an iterative optimization algorithm that employs the analytical gradient of the log-likelihood function and approximates the integrals in question by applying an Adaptive Gaussian Quadrature approach (AGH, Tuerlinckx et al., 2006). We refer the reader to Nestler (2020) for more information about the ML estimator, its derivation, and its algorithmic implementation.

### Lasso E-MELS and E-MELS Trees

Similar to the longitudinal mixed-effect model, the E-MELS bears the risk of overfitting and it assumes that the true data-generating process is linear. To address the overfitting problem, we suggest that the model be combined with Lasso Regression by subtracting the Lasso penalty for the fixed-effect vector $\varvec{\beta }$ from the log-likelihood function of the E-MELS:

20 $$\begin{aligned} \text {logL}_{\uplambda ,E-MELS}(\varvec{\theta }) = \text {logL}_{E-MELS}(\varvec{\theta }) - \uplambda \sum _{j = 1}^{p} |\beta _{j}| . \end{aligned}$$

Again, $\uplambda \ge 0$ is the regularization parameter. The goal is to estimate the fixed effects, $\varvec{\beta }$, $s_{0}$, $r_{0}$, and the parameters in $\varvec{\Phi }$ while some of the fixed-effect coefficients are shrunken to exactly zero. Let $\varvec{\gamma }$ contain $s_{0}$, $r_{0}$ and the parameters in $\varvec{\Phi }$. To optimize the log-likelihood function of the Lasso E-MELS, we suggest that a block coordinate gradient descent method that is similar to an algorithm used by Schelldorfer et al. (2014) be used to estimate the parameters of a generalized linear mixed model that was combined with a Lasso penalty. The basis of the algorithm is to cycle through the components of the full parameter vector $\varvec{\theta } = (\varvec{\beta },\varvec{\gamma })$, and to maximize the log-likelihood function of the E-MELS with respect to one parameter block at a time (i.e., $\varvec{\beta }$ or $\varvec{\gamma }$) while holding the other parameter block fixed. The integrals in the log-likelihood function are thereby approximated by the AGH approach mentioned above. A more detailed description of the algorithm can be found in “Appendix B.”

A critical step in performing the Lasso E-MELS (and Lasso Regression in general) is to choose a value for $\uplambda$. Two approaches may be employed for this: (a) an information criterion that takes model complexity into account, such as the Akaike information criterion (AIC) or the Bayesian information criterion (BIC; see Hastie et al., 2009, for an introduction), or (b) cross-validation. Here, we use the AIC and the BIC defined by

21 $$\begin{aligned} AIC_{\lambda }&= -2 \cdot \text {logL}_{\uplambda ,E-MELS}(\varvec{\theta }) + 2\cdot \text {df}_{\lambda } \nonumber \\ BIC_{\lambda }&= -2 \cdot \text {logL}_{\uplambda ,E-MELS}(\varvec{\theta }) + \text {log}(n)\cdot \text {df}_{\lambda } \end{aligned}$$

where $n = \sum _{i=1}^{I} T_i$. For $\text {df}_{\lambda }$, we suggest that $\text {df}_{\lambda } = p_{\beta \ne 0} + \text {dim}(\varvec{\Phi })$ be used. Here, $p_{\beta \ne 0}$ is the number of *nonzero* fixed effects (see Hastie et al., 2009), and $\text {dim}(\varvec{\Phi })$ is the number of estimated covariance parameters (see Bates, 2010). Our use of the information criteria is based on findings by Schelldorfer et al. 2014 that both performed well for the Lasso generalized linear mixed model. They also perform well for the related model class of regularized structural equation models (see, e.g., Scharf & Nestler, 2019).

To combine the E-MELS with regression trees, one can use an approach that is similar to the one suggested for the mixed-effect model tree (see Sela & Simonoff, 2012). Specifically, the algorithm iterates through two steps: First, given $\varvec{\hat{\nu }}_i$ for all individuals in the training sample, a cross-sectional tree can be fit for the residuals of the E-MELS (see Equation (7)). Second, given that the tree is known, dummy variables can be used to represent the tree in the E-MELS, and the above described E-MELS algorithm can be used to obtain an estimate of the elements in $\varvec{\gamma }$. These steps can be iterated until the log-likelihood of the E-MELS has converged. Note that in principle, fitting the tree in Step 1 of the algorithm can be achieved with any tree algorithm that has been proposed (Kuhn & Johnson, 2013; Hastie et al., 2009). We use the CART (classification and regression tree) algorithm as implemented in the R package rpart in our illustration, which selects split variables and split points based on the sum of the squared prediction errors. An alternative would be to employ conditional inference trees (Hothorn et al., 2006; Kuhn & Johnson, 2013) that use significance tests (i.e., their *p* values) to select the split variables and split points. However, as we do not know how this approach performs in the case of hierarchical data, we opt for the CART algorithm (see the real-data example section for details on the implementation of the algorithm).

## Illustration I: Real-data example

We will now demonstrate the models we suggested above with a first example that uses real data from the FLIP study. FLIP is a daily diary study in which about 100 individuals were asked to complete state measures of personality, affect, and motivation for 82 consecutive days. Prior to the experience sampling part of the study, all participants filled out a number of additional measures referring to affect, life satisfaction, personality, etc. The goal of the following analyses is to use these measures to forecast a person’s nervousness on the next day.

### Sample, Variables, and Data Preparation

We first describe the sample and the variables that we used in the different models. We also briefly explain how we prepared the data.

***Sample*** For this illustration, we used data from *I* = 85 participants whose average number of completed state surveys was *M* = 76.9 (*SD* = 7.14, *Min* = 33, *Max* = 82). There is little guidance on how to split the sample into a training and a test sample. Here, we split the sample into $I_1$ = 64 individuals (about 75%) and $I_2$ = 21 individuals. The observations of the first group of individuals for all time points except the last were used as the *training data* for all models. All other data was used as *test data* to evaluate predictive performance.

***Variables and data preparation*** We used the nervousness ratings as the *outcome variable*. For these ratings, participants were asked to retrospectively appraise their behavior each day with regard to the item “I was nervous.” Ratings had to be made on a 6-point Likert-type scale ranging from 1 (*not at all*) to 6 (*very*). We did not transform the data from this variable prior to the computations. A number of time-varying and time-constant predictors were included in the prediction models. Time-varying predictors were participants’ daily ratings of the adjectives sociable, creative, friendly, organized, and self-esteem. These items were measured each day, and the ratings had to be made on the same 6-point Likert-type scale as the nervousness ratings. In addition, we included the day of the week (i.e., Monday, Tuesday, etc.), the temperature on a specific day (in Celsius), and the amount of rainfall (in milliliters). We person-mean-centered the ratings of the five adjectives prior to the analyses. The person means were included in the models as time-constant predictors. We also included gender, age, trait measures of positive and negative affect (measured with three and four items, respectively, see Watson et al., 1988), life satisfaction (measured with five items, see Glaesmer et al., 2011), and participants’ values on the power motive, achievement motive, affiliation motive, intimacy motive, and fear motive (each assessed with three items, see Schönbrodt & Gerstenberg, 2012). For the Lasso mixed-effect model and the Lasso E-MELS, we z-standardized the 23 predictors prior to computing the two models. In the case of the time-varying variables, standardization was performed after we person-mean centered the variables. We used the standard deviation of each variable’s values across all participants for standardization.

### Models

In a first step, we fit the longitudinal mixed-effect model and the E-MELS to the data of the *whole* sample. This shows us the results that would be obtained, regardless of any predictions, if the covariance structure of the data is made more complex. For better comparability, we estimated the mixed-effect model with a first-order autocorrelation structure. The model was estimated using the R package nlme and the lme function (Pinheiro et al., 2020). To ease comparisons with the E-MELS, we used an ML approach (not the restricted maximum likelihood default) to obtain the model parameters. The parameters of the E-MELS were obtained with an ML approach (see above). The integrals were approximated by an AGH procedure using 10 quadrature points. An R package (called mels) was programmed for the estimation. We provide the R codes in the accompanying OSF project (https://osf.io/53scf/).

Six models were fit to the training data to build the prediction model. The first model was a longitudinal mixed-effect model with a first-order autocorrelation structure, and the second model was the E-MELS. Both were implemented as described above. The third model was a longitudinal mixed-effect model with a Lasso penalty. This model was estimated with the package glmmLasso and the function with the same name (Groll, 2017). We used the BIC to select the optimal lambda value for this model, because using the BIC is recommended by the authors of the package. Specifically, the model was fit to a grid of 50 lambda values ranging from 0 to 500, and the lambda with the smallest BIC was selected as the optimal value. To accelerate convergence, the resulting coefficients from the prior lambda value were used as starting values for the next run. We note that glmmLasso does not allow an autocorrelation parameter to be incorporated into the model and this may affect the selection of the regularization parameter. This should be considered when interpreting the results of the model. To obtain the predicted values for the selected model, we fitted a standard mixed-effect model including only the predictors with nonzero coefficients. This procedure is recommended in the literature (see, e.g., Groll & Tutz, 2014; Schelldorfer et al., 2011) as it provides more unbiased parameter estimates (especially for the variance components).

The fourth model was the Lasso E-MELS. The model was estimated using the ML algorithm described in the introduction. It is yet unknown whether model selection based on the AIC versus BIC performs better for determining the optimal lambda value, which is why we report the results based on both criteria. We determined $\lambda$ with the same procedure as described for the Lasso mixed-effect model. The predicted values were also obtained in the same way as described for the Lasso mixed-effect model, with the exception that we estimated an E-MELS instead of the standard mixed-effect model. The fifth model was a RE-EM tree that we fit with the REEMtree function from the REEMtree package (Sela & Simonoff, 2011). This function alternates between estimating a tree and estimating a mixed-effect model until it converges. In REEMtree, the tree is estimated using the rpart function that implements the CART algorithm (Therneau & Atkinson, 2019), and the growth model is estimated with the lme function. For the tree algorithm, we used the default values of REEMtree, that is, the complexity parameter was defined to be at least $c_p = 0.001$, and the minimum number of observations that must exist within a node for a split was set to at least 20. After the initial tree was formed, it was pruned by 10-fold cross-validation. Finally, the sixth model was an E-MELS tree. To determine this model, we proceeded analogously to the REEMtree function with the difference that the algorithm alternated between estimating the tree and estimating the E-MELS instead of the longitudinal mixed-effect model. When estimating the tree, we used the same default values as described for the RE-EM tree. We have not encountered any convergence problems for any of the six models. All functions needed to compute the models and the data are available in the accompanying OSF project (see https://osf.io/53scf/).

### Predictive Performance

We used each model’s result to forecast the individuals’ nervousness during their last available observation (i.e., a one-step-ahead forecast). For the 64 individuals in the training sample, we used Equation (4) to compute the Task 1 forecast. In the case of the E-MELS, we first computed a person-specific autocorrelation parameter using Equation (14) by employing the empirical Bayesian estimate of $\iota _i$ (see Hedeker & Nordgran, 2013; Skrondal & Rabe-Hesketh, 2009), for the formulas). The second group of individuals was used to obtain forecasts for prediction Tasks 2 and 3. For Task 2, we used the fixed-effect estimates obtained in the training sample to forecast the last observation for each of the 21 individuals. For Task 3, we used all observations of the 21 individuals except the last along with the model parameter to obtain the random effect estimates for these individuals. The resulting values were then used with Eq. (4) and/or Eq. (14) (in the case of the E-MELS models) to predict the last observation. We used the mean squared error (MSE) to compare the predictive accuracy of the models. The MSE was obtained by computing the squared differences between the observed values and the model’s predictions. Then, the mean of these squared residuals was computed. As a measure of predictive precision, we also calculated the average standard deviation of the forecast errors. To this end, we first used Eq. (10) or Eq. (12) to obtain a variance estimate for the error of a specific forecast. After taking the root of the resulting value, we averaged these standard deviations for each model and prediction task. We do not report the forecast error standard deviation for the mixed-effect model tree or the E-MELS tree, because Eqs. (10) and (12) cannot be used for these two models and, to our knowledge, there is no established approach to obtain the forecast error variance (or standard deviation) for trees in case of hierarchical data.

### Results

Table 1 shows the parameter estimates of the standard longitudinal mixed-effect model and the standard E-MELS. The table also contains the log-likelihood values, the AIC, and the BIC. The fixed-effect coefficient estimates were very similar across the two models such that seven predictors had weights that were significantly different from zero when we applied the conventional significance value of .05. Comparing the longitudinal mixed-effect model with the E-MELS showed that people differed in the amounts of within-person variance they had. The mean variance estimate was exp(0.06) = 1.07, and the variance of the within-person variance terms was $\phi ^2_{\omega }$ = 0.20. Interestingly, there were only minimal between-person differences with regard to the autocorrelation parameter: The mean autocorrelation parameter was atanh(0.19) = 0.19, and the variance was $\phi ^2_{\iota }$ = 0.02. A likelihood-ratio test showed that the E-MELS fit the data better than the standard longitudinal mixed-effect model, $\chi ^2 = 479.5$, $df = 5$, $p < 0.01$.

In the second step, we estimated the prediction models using the training data and then evaluated the predictive performance of the models using the test data. Table 2 shows the MSE and the average standard deviation of the forecast errors (called $\hat{\sigma }_F$) for the six models for the three different prediction tasks. For all methods, the MSE was lowest when we used the model for the task of predicting the nervousness of persons for whom past observations are available and these persons were also used to determine the prediction model (i.e., Task 1). The MSE was comparable for the two other two tasks (i.e., Task 2 and Task 3). The standard deviation of the forecast errors was lower for the two tasks which used past observations of the persons to compute the forecast (i.e., Task 1 and Task 3) compared to the prediction task which did not use prior observations (i.e., Task 2).

**Fig. 2:**
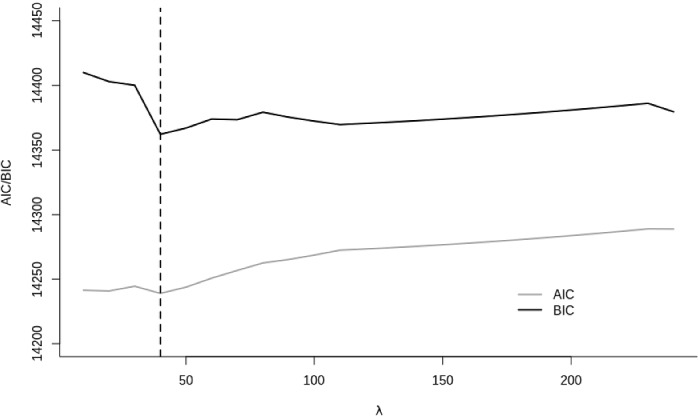
Results for the AIC/BIC of the E-MELS Lasso for the training data as a function of the regularization parameter $\uplambda$.

As can be seen in Table 2, the predictive accuracy (i.e., the MSE) of the mixed-effect model hardly differed from the accuracy of the E-MELS. Thus, the additional consideration of interindividual differences in within-person variance and within-person autocorrelation did not seem to improve the predictions when we compare these two models. Also, the predictive accuracy of the mixed-effect Lasso did not differ from the performance of the former two models. The regularization parameter associated with this model was $\uplambda$ = 110. Furthermore, the weights of three predictors were set to zero (see Table 2), the log-Likelihood was -7270.92, and the BIC was 14745.5. For the E-MELS Lasso, the regularization parameter was $\uplambda$ = 40 when we use the BIC for model selection. When we used the AIC (see Fig. 2 for a plot of the BIC or the AIC, respectively, against $\uplambda$), the regularization parameter was also $\uplambda$ = 40. The selected model contained twelve predictors (see Table 2). The log-Likelihood of this model was $-7096.99$, the AIC was 14235.9, and the BIC was 14372.2. With regard to the accuracy of the predictions, we found that this model showed the worst performance for Task 1. For Tasks 2 and 3, the performance was similar to the mixed-effect model, the E-MELS, and the mixed-effect Lasso. Finally, the results concerning predictive precision showed that the average standard deviation of the forecast errors was lower for both E-MELS models as compared to the two mixed-effect models.

With regard to the two models based on trees, we found that the mixed-effect model tree resulted in 24 terminal nodes and a log-Likelihood value of $-7222.28$. For prediction Task 1, the predictive performance in terms of accuracy of this tree was similar to the performance of the other models. However, worse performance was obtained for the second and the third task (see “Appendix C” for a plot of the estimated E-MELS tree). The log-Likelihood value of the tree was $-7032.93$, it consisted of seventeen terminal nodes, and negative affect, self-esteem, friendliness, creativeness, organized, and daily temperature were predictive of daily nervousness. Finally, the predictive performance of the E-MELS tree was better than the performance of mixed-effect model tree for all three types of prediction tasks. Compared with the remaining models, its performance was worse for Task 2 but better for Task 3.

**Table 1:** Results for the real-data example across four models.

	Mixed-effect model		E-MELS	
	Standard	Lasso	Standard	Lasso
*Predictors*				
Intercept	1.59 (0.71)	1.29	1.72 (0.68)	1.72
Age	0.01 (0.01)	0.02	0.01 (0.01)	–
Sex	0.11 (0.16)	0.21	0.11 (0.15)	–
Positive affect	$-0.24$ (0.18)	$-0.43$	$-0.21$ (0.16)	0.03
Negative affect	**0.23** (0.08)	0.12	**0.22** (0.08)	0.28
Life satisfaction	0.08 (0.06)	0.02	0.05 (0.06)	$-0.01$
Power	0.03 (0.07)	0.05	0.03 (0.06)	–
Achievement	0.07 (0.07)	0.17	0.03 (0.07)	–
Affiliation	0.29 (0.27)	0.64	0.29 (0.26)	–
Intimacy	$-0.03$ (0.08)	$-0.09$	0.01 (0.08)	–
Fear	$-0.04$ (0.07)	$-0.09$	$-0.05$ (0.07)	–
Sociable (PM)	$-0.23$ (0.14)	$-0.27$	$-0.21$ (0.14)	–
Creative (PM)	**0.36** (0.09)	0.40	**0.32** (0.09)	0.31
Friendly (PM)	0.16 (0.17)	0.44	0.09 (0.17)	–
Organized (PM)	0.00 (0.13)	$-0.17$	0.02 (0.13)	–
Self-esteem (PM)	$-\mathbf{0}.46$ (0.11)	$-0.53$	$-\mathbf{0}.38$ (0.10)	$-0.37$
Sociable	**0.03** (0.02)	–	**0.05** (0.01)	0.04
Creative	0.01 (0.02)	$-0.01$	0.01 (0.01)	–
Friendly	$-0.04$ (0.02)	$-0.01$	$-0.03$ (0.02)	$-0.02$
Organized	**0.05** (0.02)	0.05	**0.05** (0.02)	0.06
Self-esteem	$-\mathbf{0}.16$ (0.02)	$-0.17$	$-\mathbf{0}.15$ (0.02)	$-0.16$
Weekday	$-\mathbf{0}.04$ (0.01)	$-0.04$	$-\mathbf{0}.04$ (0.01)	$-0.03$
Temperature	0.00 (0.01)	–	0.01 (0.00)	0.01
Amount rainfall	0.01 (0.01)	–	0.01 (0.00)	0.01
*Intercept Variance*	0.20	0.18	0.21 (0.04)	0.23
*Residual Variance*				
Intercept	1.23	1.19	1.07 (1.05)	1.02
Variance	–	–	0.20	0.20
*Autocorrelation*				–
Intercept	0.18	0.20	0.19 (0.02)	0.21
Variance	–		0.02	0.02
log-Likelihood	$-9946.19$	$-7270.92$	$-9$706.46	$-70$96.99
AIC	19946.40	14589.84	19476.92	14235.98
BIC	20129.58	14745.50	19694.02	14372.18

PM = Person mean. For the fixed-effect coefficients, values in parentheses denote standard errors and coefficients in bold are significant ($p < .05$). Not shown are the covariance parameters between the random effects. For the Lasso models, we show the parameters of the selected model for the *training* data. Note that we do not report standard errors for these parameters as these are not trustworthy. For the Lasso mixed-effect model, the regularization parameter was $\lambda$ = 110 and for the Lasso E-MELS the parameter was $\lambda$ = 40.

**Table 2:** Results concerning the mean squared error (MSE, standard errors in parentheses) and the standard deviation of the forecast error ($\hat{\sigma }_F$) of a one-step-ahead forecast across the six models for the real-data example.

		Mixed-effect model			E-MELS		
Measure	Task	Standard	Lasso	Tree	Standard	Lasso	Tree
MSE	1	0.84 (0.15)	0.85 (0.15)	0.82 (0.13)	0.89 (0.15)	0.94 (0.16)	0.90 (0.17)
	2	1.37 (0.35)	1.41 (0.36)	2.61 (0.79)	1.40 (0.36)	1.42 (0.37)	1.60 (0.41)
	3	1.41 (0.37)	1.41 (0.37)	2.20 (0.63)	1.37 (0.34)	1.40 (0.35)	1.16 (0.31)
$\hat{\sigma }_F$	1	1.08	1.08	–	1.04	1.04	–
	2	1.18	1.18	–	1.13	1.13	–
	3	1.08	1.09	–	1.04	1.03	–
Computation Time (in sec)		2.71	18.61	9.41	131.9	2926.5	1100.9

Task 1 refers to predictions for a new time point for persons that were used to build the prediction model. Task 2 refers to predictions for new persons without considering prior data, and Task 3 refers to predictions for new persons with considering prior data.

In summary, the standard models showed the best predictive performance in terms of accuracy across all three tasks. Altogether, however, the predictive power of the models was low to moderate, which may indicate that the predictors used in this example were not sufficient to comprehensively predict people’s daily nervousness. Another explanation could be that we used a single item as the outcome variable, so that the low predictive performance might be due to measurement error. However, an intra-class correlation coefficient of 0.27 suggests that at least some of the between-person differences in nervousness are reliably measured with the item. The generally low predictive accuracy could also explain why the consideration of interindividual differences in the level-1 variance and in the autocorrelation did not lead to a stronger improvement in the predictive performance. Another explanation could be that the individuals only slightly differed with regard to their autocorrelation, which directly affects the forecast (see Eq. 4). It is possible that stronger effects on predictive accuracy would be observed in data with larger differences between the subjects. Finally, we found—at least when we compared the standard models with the Lasso models—that the consideration of interindividual differences in the autocorrelation and in the Level 1 residual variance led to more precise forecasts.

## Illustration II: Simulated-data example

In the real-data example, there were little differences in predictive performance between the six approaches. We now report the results for a simulated-data example to provide a clearer demonstration of the differences between the models.

### Data and Sample

We simulated data including nine predictors. Three predictors were related to the outcome variable and six predictors were not associated with the outcome. All predictors were drawn as independent, uniformly distributed random variables on the interval $\left[ 0, 10 \right]$. Using the three relevant predictors, we generated an outcome variable that conforms to a regression tree with four leaves. We used the following rules to generate the tree (see Hajjem et al. 2011, for a similar approach):

22 $$\begin{aligned} \text {Leaf 1: If }&x_{1it} \le 5 \text { and } x_{2it} \le 5 \text {, then } y_{it} = 10 + \tau _i + \epsilon _{it} \nonumber \\ \text {Leaf 2: If }&x_{1it} \le 5 \text { and } x_{2it}> 5 \text {, then } y_{it} = 11 + \tau _i + \epsilon _{it} \nonumber \\ \text {Leaf 3: If }&x_{1it}> 5 \text { and } x_{3it} \le 5 \text {, then } y_{it} = 12 + \tau _i + \epsilon _{it} \nonumber \\ \text {Leaf 4: If }&x_{1it}> 5 \text { and } x_{3it} > 5 \text {, then } y_{it} = 13 + \tau _i + \epsilon _{it} \end{aligned}$$

where $\epsilon _{it}$ is an element of person *i*’s Level 1 residual term vector $\varvec{\epsilon _i}$. This vector was drawn from a multivariate normal distribution (see Equations (13) and (14)) with $s_0$ set to $-0.67$ and $r_0$ set to 0.26. Furthermore, the covariance matrix of the random effect terms, $\tau _i$, $\omega _i$, and $\iota _i$ was a 3 x 3 matrix with diagonal elements 1.0, 0.5, and 0.5. The off-diagonal elements were set to zero (i.e., the random effects were uncorrelated). Thus, in this example, persons differ in their levels, in the within-person variance, and in the autocorrelation.

We generated data for $I = 200$ participants with $T_i = 51$ time points for all *i*. The first 50 measurements for the first 100 persons were used as the training sample. We used the remaining time points and persons to measure the predictive performance of a one-step-ahead forecast for the three prediction tasks in an analogous way as in the real-data example.

### Results

We fitted the same six models with the same model specifications as in the real-data example. For the mixed-effect model, the Lasso mixed-effect model, the E-MELS, and the E-MELS Lasso, we not only included the nine predictor variables but also interaction terms between the first and the second and the first and the third predictor variables, because these second-order terms are represented in the tree. This allows us to examine whether the Lasso model selects the correct variables.

**Fig. 3:**
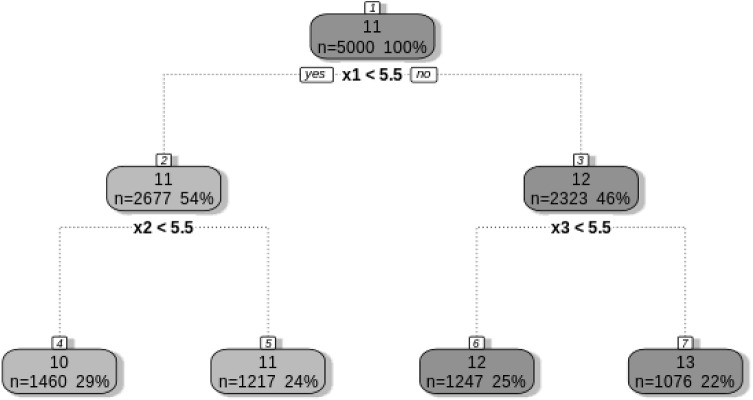
Estimated E-MELS tree for the simulated training data.

Figure 3 shows that the E-MELS tree accurately detected the four terminal nodes and that it selected the correct splitting variables and split points. The same result emerged for the mixed-effect model tree. Table 3 presents the parameter estimates for the non-tree models. The E-MELS Lasso (using the AIC) and the mixed-effect Lasso kept the correct number of predictor variables. With regard to predictive accuracy (see Table 4), we found that the MSE was lower for the E-MELS approaches for prediction Tasks 1 and 3 but not for Task 2. However, and as expected, the best performance was obtained by the E-MELS tree. Furthermore, we also found that the average standard deviation of the forecast errors was lower for the standard E-MELS and the Lasso E-MELS, as compared to the two respective mixed-effect models. Thus, accounting for interindividual differences in the within-person variance and in the autocorrelation can indeed significantly improve the quality of predictions.

## Discussion

The increasing availability of intensive longitudinal data has led to a number of methodological articles in which the longitudinal mixed-effect model was combined with some classic machine-learning approaches such as Lasso regression and regression trees (e.g., Fan & Li, 2012; Hajjem et al., 2011; Li et al., 2018; Schelldorfer et al., 2011, Sela & Simonoff 2012). In the present article, we extended these earlier approaches by suggesting a combination of the E-MELS (a variant of the mixed-effect location-scale model, e.g., Hedeker et al., 2008; Hedeker et al., 2009; Nestler, 2020; 2021) with a Lasso penalty and a regression tree. In contrast to the earlier models, in addition to the level, our extensions also allow individuals to have differences in the within-person variance and the autocorrelation. Besides the description of the models, we also explained how to estimate the models, we implemented all the algorithms in R, and we illustrated how to use them by applying them to a real data and a simulated data set.

One motivation for combining the models was that by considering the three sources of interindividual differences (i.e., the intercepts, the residual variance, and the autocorrelation), the predictive performance would improve in comparison with the other models. This should be especially true when predicting values for people for whom longitudinal data are available and considered in the prediction (in contrast to individuals for whom no past observations are available). The results of the simulated (and partly the real) data example and a further simulation (see “Appendix A”) supported our assumption by showing that the E-MELS and its extension had a better predictive performance than the other approaches that did not incorporate all three sources of variance. One interesting observation in our illustrative data example was that the between-person variance of the autocorrelation was very small compared to the residual variance. We find this result very interesting from a psychological perspective because, in the more applied literature, the variance and the autocorrelation are considered two important components of a person’s stability (Jahng et al., 2008; Ram & Gerstorf, 2009). At present, however, it is unclear whether the two components are equally important and how independent these two components actually are. We believe that future research should investigate this question more closely by using the E-MELS, as this model allows for estimating the amount of between-person differences in the two stability components and their relationship (e.g., the covariance $\phi _{\omega \iota }$).

**Table 3:** Results for the simulated-data example across four models.

	Mixed-effect model		E-MELS	
	Standard	Lasso	Standard	Lasso
*Predictors*				
Intercept	9.11	9.07	9.18	9.18
$x_1$	0.31	0.31	0.30	0.30
$x_2$	0.21	0.21	0.19	0.19
$x_3$	$-0$.04	−0.04	$-0.04$	$-0.04$
$x_4$	$-0$.01	–	$-0.01$	–
$x_5$	0.01	–	0.01	–
$x_6$	0.01	–	0.01	–
$x_7$	0.01	–	0.01	–
$x_8$	$-0$.01	–	$-0.01$	–
$x_9$	$-0$.01	–	0.01	–
$x_1 x_2$	−0.04	$-0.03$	$-0.02$	$-0.02$
$x_1 x_3$	0.02	0.02	0.02	0.03
*Intercept variance*	1.13	1.13	0.94	0.94
*Residual variance*				
Intercept	1.70	1.70	1.08	1.08
Variance	–	–	0.20	0.20
*Autocorrelation*				
Intercept	0.25	0.25	0.22	0.22
Variance	–	–	0.27	0.28

Not shown are the standard errors of the parameters and the covariance parameters between the random effects. For the Lasso models, we show the parameters obtained with the final model in which the outcome was fitted to the selected variables. For the Lasso mixed-effect model, the regularization parameter was $\lambda$ = 320 and for the Lasso E-MELS the parameter was $\lambda$ = 220.

**Table 4:** Results concerning the mean squared error (MSE, standard errors in parentheses) and the standard deviation of the forecast error ($\hat{\sigma }_F$) of a one-step-ahead forecast across the six models for the simulated-data example.

		Mixed-effect model			E-MELS		
Measure	Task	Standard	Lasso	Tree	Standard	Lasso	Tree
MSE	1	1.44 (0.24)	1.45 (0.25)	1.01 (0.20)	1.21 (0.22)	1.20 (0.22)	0.81 (0.14)
	2	2.61 (0.37)	2.59 (0.37)	2.21 (0.37)	2.62 (0.37)	2.61 (0.37)	2.22 (0.37)
	3	1.54 (0.22)	1.56 (0.22)	1.15 (0.18)	1.20 (0.17)	1.21 (0.17)	0.67 (0.10)
$\hat{\sigma }_F$	1	1.28	1.29	–	1.09	1.09	–
	2	1.65	1.66	–	1.43	1.42	–
	3	1.29	1.29	–	1.09	1.08	–
Computation time (in sec)		0.89	8.34	1.82	85.3	519.4	101.1

Task 1 refers to predictions for a new time point for persons that were used to build the prediction model. Task 2 refers to predictions for new persons without considering prior data, and Task 3 refers to predictions for new persons with considering prior data.

This would also be interesting from a more predictive standpoint. In our data examples (and the simulation), we examined whether considering between-person differences in both the residual variance and the autocorrelation improves the accuracy and the precision of the forecasts. An interesting question for future research is to find out which of the two types of individual differences is more central to a forecast’s accuracy or precision. For example, Equation 4 shows that the autocorrelation directly affects accuracy. However, person-specific variances may affect forecast accuracy indirectly, because omitting interindividual differences in the residual variance may lead to biases in the estimates of the elements in $\varvec{\Phi }$ which may in turn affect the estimates of a person’s random effects. Future research should therefore look at whether accounting for interindividual differences in autocorrelation always has stronger effects on forecasting accuracy than accounting for interindividual differences in the residual variance (similarly, but with reversed roles, this could be examined for prediction precision). Our work also raises a number of other interesting questions and topics for future methodological research. To begin with, simulation research is needed to compare the predictive performance of the suggested models in different situations. For instance, the influence of interindividual differences in the Level 1 variance and the autocorrelation on predictive performance must be investigated using different combinations of population parameters (e.g., different average autocorrelation values and different values of the autocorrelation variance). Furthermore, although modern data collection procedures allow us to measure a fair amount of longitudinal data for a large number of different individuals, we believe that it is important to investigate how many participants and measurements within a participant are necessary to make a good prediction. These simulations could also be used to investigate whether and how the reliability of the predictors influences the quality of the predictions.

Furthermore, in our extensions, we assumed that the time intervals between the measurements were equally spaced. This assumption is justified in many intensive longitudinal studies, including our example with real data, but there are also studies in which the time intervals are not equally spaced. It would be interesting to examine how such a misspecification affects the predictive performance of the models introduced here, because it may lead, among other things, to a biased estimate of the autocorrelation or the Level 1 residual variance parameter. One could also consider alternative serial correlation structures—such as the exponential serial correlation function (Diggle et al., 2002; Vansteelandt & Verbeke, 2016)—that do not rest on the assumption of equally spaced time intervals in the combined E-MELS models. Finally, another assumption we made was that $\varvec{X}_{i, T_i + H}$ is known to compute the *H*-step forecast. This assumption may be more or less justified depending on the type of predictors contained in $\varvec{X}_{i, T_i + H}$. It is usually not problematic in the case of time-constant variables that only need to be recorded once for each person and for variables coding time or seasonal variables that consist of predefined values (e.g., weekdays). For time-varying contemporaneous or lagged predictors, the assumption is more problematic, and if the values are not available for time point $T_i + H$, one has to estimate them to calculate the forecast. Different methods exist for this Hyndman & Athanasopoulos, 2018, and we think that it is interesting to investigate the extent to which they influence the magnitude of the prediction error in the E-MELS.

With regard to Lasso E-MELS, it would be interesting to combine it with other penalty terms such as the ridge penalty or the elastic net penalty (James et al., 2013; Scharf & Nestler, 2019). This might be especially interesting from a more explanatory perspective as both have been found to perform well when the predictors are highly correlated, which in turn leads to untrustworthy significance tests. Furthermore, in the suggested combinations, the penalty term referred to the fixed effects only. Therefore, another interesting task for future research would be to extend this to the random-effect covariance matrix. This extension will also be very interesting—and challenging—from a computational perspective. Finally, in our illustrative example, Lasso E-MELS was not sensitive to the information criterion used to select the regularization parameter $\lambda$. However, we believe that it would be interesting to more thoroughly examine the performance of the AIC and BIC for model selection and to further compare them to the performance of cross-validation.

Regarding the E-MELS tree, another interesting extension would be to combine the E-MELS with a random forest instead of a single regression tree (Kuhn & Johnson, 2013). Random forests usually have a better predictive performance than single regression trees in the context of cross-sectional data. We believe that it will be an interesting task for future research to examine whether this generalizes to intensive longitudinal data and the E-MELS. Finally, it would also be of great interest to compare the predictive performance of the Lasso E-MELS and the E-MELS tree to other models such as the lagged dependent variable multilevel model (Asparouhov et al., 2018).

There are also a number of interesting research questions on the algorithmic level. In our implementation, we used an AGH procedure to numerically approximate the integrals. However, the computation of this approximation can be slow, and we believe that future research should examine other approximations (e.g., the Laplace approximations) or other algorithms (e.g., a Monte Carlo EM algorithm, see Booth & Hobert, 1999, or variational approximations, see Ormerod & Wand, 2010). Alternative algorithms have also been proposed for mixed models that include a penalty term Groll & Tutz, 2014). Again, an interesting task for future research would be to implement and compare these different approaches for the Lasso E-MELS. In the case of the E-MELS tree, we used the CART algorithm to estimate the tree. However, CART suffers from several problems, such as favoring predictors with more values, that is, variables that have more distinct numerical values or variables with fewer missing values. Although this seems to be more of a problem when one uses random forests (Kuhn & Johnson, 2013), we nevertheless believe that future research should examine conditional inference trees as an alternative (Hothorn et al., 2006).

So far, all our research questions relate to the mixed-effect model and our proposed extensions. In our opinion, however, another important question for future research is to compare the predictive performance of the mixed-effect models with other forecasting models, such a time series model for a single individual. For instance, mixed-effect models may outperform individual time series models in terms of prediction in some situations, because they can take the same time-varying variables into account as a time series model (e.g., lagged variables), but in addition, they can also use time-constant and/or person-level variables. The inclusion of such further variables should improve the quality of predictions if the variables are indeed predictive of the outcome and if the predictors were sampled representatively. Another potential advantage of mixed-effect models is that they use information from all individuals to estimate the fixed effects of, for example, the time-varying predictor variables. Thus, when the data collected for a specific person is rather sparse and not representative of the person’s true stream of data, this person’s weight might still be estimated with an acceptable accuracy. However, the relative performance of mixed-effect versus time series models will depend on a number of issues (e.g., the number of time points). Nevertheless, in our opinion it is interesting to compare the models in these different data situations (see Afshartous & de Leeuw, 2005.)

To summarize, this paper proposed a combination of a regression tree and a Lasso regression with an extended version of the MELS. We described the combinations, and we showed how to estimate the model’s parameters. We believe that the described and proposed models will be of interest to applied researchers. Furthermore, our presentations raise a number of interesting research questions, the solutions of which promise further interesting model developments.

## Figures and Tables

**Fig. 4:**
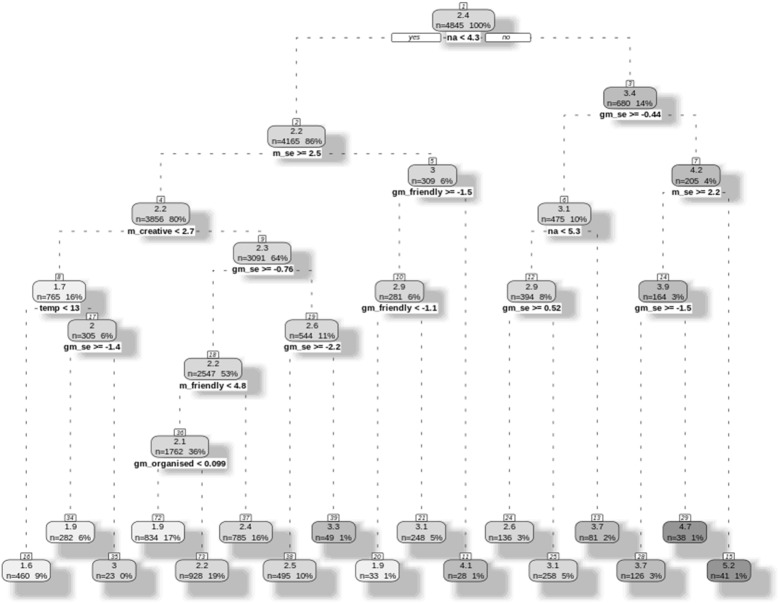
Estimated E-MELS tree for the training data with abbreviated variable names to improve readability (M = person mean, GM = person mean centered). na = negative affect, se = self-esteem, creative = creativeness, friendly = friendliness, organized = organized, temp = temperature.

## Appendix A

We conducted a small simulation study to investigate whether an extension of the mixed-effect model that considers between-person differences in the Level 1 variance and the autocorrelation (i.e., the E-MELS) yields more precise forecasts than a standard longitudinal mixed-effect model. To this end, we simulated data from a random-intercept E-MELS in which person *i*’s outcome value at time point *t* are a function of the person’s value in a time-varying variable $X_{it}$, a time-constant variable $W_{i}$, and their interaction. Specifically, the Level 1 and Level 2 equations are (see Afshartous & de Leeuw, 2005 for a similar approach):

23 $$\begin{aligned} \text {L1: } Y_{it}&= \gamma _{0i} + \gamma _{1i} X_{it} + \epsilon _{it} \nonumber \\ \text {L2: } \gamma _{0i}&= \beta _{00} + \beta _{01}W_{i} + \tau _{i} \nonumber \\ \gamma _{1i}&= \beta _{10} + \beta _{11}W_{i} \nonumber \\ \sigma _{\epsilon _i}^{2}&= \text {exp}( s_0 + \omega _i) \nonumber \\ \rho _{i}&= \text {tanh}( r_0 + \iota _i) \end{aligned}$$

where $\beta _{00}$, $\beta _{01}$, $\beta _{10}$, and $\beta _{11}$ were all set to 1. Both $X_{it}$ and $W_{i}$ were simulated as standard normal random variables. The covariance matrix of the random effect terms, $\tau _{i}$, $\omega _i$, and $\iota _i$, was

24 $$\begin{aligned} \varvec{\Phi } = \begin{bmatrix} 1.00 &{} -0.20 &{} 0.20 \\ &{} 0.50 &{} 0.10 \\ &{} &{} 0.50 \\ \end{bmatrix}. \end{aligned}$$

We set $s_0$ (= -0.67) and $r_0$ (= 0.26) in such a way that when we fit a standard mixed-effect model to data generated from this population model, the (average) Level 1 residual variance is $\sigma ^2_{\epsilon } = 1$ and the (average) autocorrelation is $\rho$ = 0.3. The ICC then approximately corresponds to 0.50, which we consider to be a typical value for intensive longitudinal data (Snijders & Bosker, 2012).

The number of persons in the training sample was held constant at 100 subjects in a replication. We varied the number of time points by drawing either 25 or 50 measurements per subject in the training sample. The *R* package mvtnorm (Genz et al., 2019) was used to generate the population and to draw 500 samples from the population in each of the two simulation conditions. Finally, a standard longitudinal mixed-effect model with a AR1 structure and the E-MELS were used to estimate the model parameters within a replication. We used the nlme package (Pinheiro et al., 2020) for the longitudinal mixed-effect model.

We examined predictive performance for the three prediction tasks mentioned in the main text. For prediction Task 1, we simulated another data point for each person in the training sample (i.e., a 26th or a 51st measurement). This true value was compared with a forecast that we calculated using Eq. (4). In the case of the E-MELS, person-specific autocorrelation parameters were computed with the empirical Bayesian estimates of a person’s random effect estimate for the autocorrelation $\hat{\iota }_i$ (see Eq. 14). For prediction Tasks 2 and 3, we generated 100 test persons in each replication with 26 or 51 measurements, respectively. For Task 2, we used the fixed-effect estimates to compute the forecast for the 26th or 51st time point, respectively, for each of the 100 test persons (i.e., we used the formula $\hat{y}_{i,H} = \varvec{X}_{i,H} \varvec{\hat{\beta }}$). For Task 3, the first 25 or 51 measurements, respectively, were used to estimate the random effects and the Level 1 residuals for the 100 test persons given the parameter estimates of the training sample. We then used Eq. (4) and/or Eq. (14) to compute the forecast and Eq. (10) to compute the standard deviation of the forecast error (i.e., the square root of a forecast error’s variance; Frees, 2004, Chapter 4).

**Table 5:** Average parameter estimates depending on number of measurements (*T*).

*T*	$\bar{\beta }$	$\phi ^2_{\tau }$	$\phi ^2_{\omega }$	$\phi ^2_{\iota }$	$s_0$	$r_0$
25	0.999	0.981	0.497	0.493	$-0.655$	0.271
50	1.002	0.982	0.506	0.490	$-0.654$	0.265

$\bar{\beta }$ is the average across all four fixed-effect coefficients (that were all set to 1).

For all three predictions tasks, 100 forecasts are available in each replication. We computed the mean squared error (MSE) of these one-step-ahead forecasts to have a measure of prediction accuracy. Specifically, the MSE was computed by taking the squared difference of the forecast and the true outcome value. We then computed the average of these values across all replications in the respective simulation condition. As a measure of prediction precision, we used the average of the forecast errors’ standard deviations across all replications in the respective simulation condition ($\hat{\sigma }_F$). Finally, we also computed the average parameter estimates across the 500 replications to examine the performance of the E-MELS with regard to estimating the population parameters.

**Table 6:** Mean squared error (MSE) of prediction and standard deviation of the forecast error ($\hat{\sigma }_F$) for a one-step forecast depending on the model and the number of training time points (*T*).

		Mixed-Effect Model			E-MELS		
Measure	*T*	Task 1	Task 2	Task 3	Task 1	Task 2	Task 3
MSE	25	1.123	2.526	1.183	0.840	2.521	0.871
	50	1.139	2.407	1.176	0.805	2.403	0.832
$\hat{\sigma }_F$	25	1.099	1.561	1.101	1.019	1.250	1.016
	50	1.102	1.554	1.107	1.020	1.249	1.023

Task 1 refers to predictions for persons that were used to build the prediction model, Task 2 refers to predictions for new persons without considering prior data, and Task 3 refers to predictions for new persons with considering their prior data.

Table 5 shows that the E-MELS estimates were very close to the true population parameters. Table 6 reports the models’ MSE and $\hat{\sigma }_F$ for the three prediction tasks. In both conditions, the MSE for the E-MELS was lower than the MSE for the mixed-effect model for Task 1 and Task 3. Almost no differences occurred for prediction Task 2. An explanation for the latter result is that the fixed effects were also estimated in the standard model with little bias: The average estimate was 0.999 in the case of 25 and 1.001 in the case of 50 measurements (true $\beta$s = 1). However, the intercept variance was strongly biased in the standard model (true $\phi _{\tau }^2$ = 1). The average estimate was 1.339 for 25 measurements and 1.218 for 50 measurements. With regard to $\hat{\sigma }_F$, the value was close to the simulated (average) Level 1 residual variance in case of the E-MELS for Task 1 and Task 3. For the mixed-effect model, this value was almost 0.10 units higher. A larger difference was found for Task 2, which again could be explained by the biased estimation of the intercept variance.

## Appendix B

In this appendix, we briefly describe the algorithm for the Lasso E-MELS. Specifically, we used an adapted version of an algorithm (called Approximate GLMMLasso) proposed by Schelldorfer et al. 2014. To obtain the estimates of $\varvec{\theta } = (\varvec{\beta },\varvec{\gamma })$, we minimize the following function:

$$\begin{aligned} Q^{AGH}_{\uplambda } = -2 \text {logL}_{E-MELS}(\varvec{\theta }) + \uplambda \sum _{j = 1}^{p} |\beta _{j}| = f(\varvec{\theta }) + \uplambda \sum _{j = 1}^{p} |\beta _{j}| \end{aligned}$$

where the superscript *AGH* indicates that the integrals in *f* are approximated by AGH quadrature. Furthermore, let $\varvec{e}_j$ be the *j*th unit vector, *s* the *s*th iteration step, and $\varvec{\theta }^{(s)}$ the parameters in this step. Furthermore, for the coefficients in $\varvec{\beta }$ we use this notation:

$$\begin{aligned} \varvec{\beta }^{(s,s-1,\beta _k)}&= (\beta _{1}^{(s)}, \cdots , \beta _{(k-1)}^{(s)}, \beta _{k}, \beta _{k+1}^{(s-1)}, \cdots , \beta _{p}^{(s-1)} ) \\ \varvec{\beta }^{(s,s-1;k)}&= (\beta _{1}^{(s)}, \cdots , \beta _{(k-1)}^{(s)}, \beta _{k}^{(s-1)}, \beta _{k+1}^{(s-1)}, \cdots , \beta _{p}^{(s-1)} ). \end{aligned}$$

The algorithm is given in Algorithm 1.

## Appendix C

See Fig. 4

## Footnotes

1 We assume that the data was collected in a representative way, so that it covers a large range of (combinations of) the predictor values that might occur in forecasting tasks.

2 In the mixed-effect model literature, this predictor is called the Best Linear Unbiased Predictor (BLUP) and its derivation presumes that the variance components of the longitudinal mixed-effect model are known. In real-data contexts, the maximum likelihood estimates, for example, can be used to compute the BLUP; the resulting prediction is called the empirical BLUP (EBLUP, see Frees, 2004; Searle et al., 1992; Skrondal & Rabe-Hesketh, 2009).

## References

[CR1] Afshartous D, de Leeuw J (2005). Prediction in multilevel models prediction in multilevel models. Journal of Educational and Behavioral Statistics.

[CR2] Asparouhov T, Hamaker EL, Muthén BO (2018). Dynamic structural equation models dynamic structural equation models. Structural Equation Modeling: A Multidisciplinary Journal.

[CR3] Baird BM, Le K, Lucas RE (2006). On the nature of intraindividual personality variability: Reliability, validity, and associations with well-being. Journal of Personality and Social Psychology.

[CR4] Baird BM, Lucas RE, Donnellan MB (2017). The role of response styles in the assessment of intraindividual personality variability. Journal of Research in Personality.

[CR5] Bates, D. M. (2010). lme4: Mixed-effects modeling with R. http://lme4.r-forge.r-project.org/book/.

[CR6] Booth JG, Hobert JP (1999). Maximizing generalized linear mixed model likelihoods with an automated Monte Carlo EM algorithm. Journal of the Royal Statistical Society: Series B (Statistical Methodology).

[CR7] Chi EM, Reinsel GC (1989). Models for longitudinal data with random effects and AR(1) errors. Journal of the American Statistical Association.

[CR8] Diggle P, Heagerty P, Liang K-Y, Zeger S (2002). Analysis of longitudinal data.

[CR9] Fan Y, Li R (2012). Variable selection in linear mixed effects models. Annals of Statistics.

[CR10] Frees EW (2004). Longitudinal and panel data.

[CR11] Fu W, Simonoff JS (2015). Unbiased regression trees for longitudinal and clustered data. Computational Statistics and Data Analysis.

[CR12] Gasimova F, Robitzsch A, Wilhelm O, Hülür G (2014). A hierarchical Bayesian model with correlated residuals for investigating stability and change in intensive longitudinal data settings. Methodology.

[CR13] Genz, A. , Bretz, F. , Miwa, T. , Mi, X. , Leisch, F. , Scheipl, F., & Hothorn, T. (2019). mvtnorm: Multivariate normal and t distributions [Computer software manual]. https://CRAN.R-project.org/package=mvtnorm (R package version 1.0-11).

[CR14] Geukes K, Nestler S, Hutteman R, Dufner M, Kuefner ACP, Egloff B, Back MD (2017). Puffed up but shaky selves: State self-esteem level and variability in narcissists. Journal of Personality and Social Psychology.

[CR15] Geukes K, Nestler S, Hutteman R, Kuefner ACP, Back MD (2017). Trait personality and state variability: Predicting individual differences in within-and cross-context fluctuations in affect, self-evaluations, and behavior in everyday life. Journal of Research in Personality.

[CR16] Glaesmer H, Grande G, Braehler E, Roth M (2011). The German version of the satisfaction with life scale (SWLS). European Journal of Psychological Assessment.

[CR17] Goodfellow I, Bengio Y, Courville A (2016). Deep learning.

[CR18] Groll, A. (2017). glmmLasso: Variable selection for generalized linear mixed models by L1-penalized estimation [Computer software manual]. https://CRAN.R-project.org/package=glmmLasso R package version 1.5.1.

[CR19] Groll A, Tutz G (2014). Variable selection for generalized linear mixed models by L1-penalized estimation. Statistical Computing.

[CR20] Hajjem A, Bellavance F, Larocque D (2011). Mixed effects regression trees for clustered data. Statistics and Probability Letters.

[CR21] Hamaker EL, Asparouhov T, Brose A, Schmiedek F, Muthén B (2018). At the frontiers of modeling intensive longitudinal data: Dynamic structural equation models for the affective measurements from the COGITO study. Multivariate Behavioral Research.

[CR22] Harlow LL, Oswald FL (2016). Big data in psychology: Introduction to the special issue. Psychological Methods.

[CR23] Hastie T, Tibshirani R, Friedman J (2009). The elements of statistical learning.

[CR24] Hedeker D, Demirtas H, Mermelstein RJ (2009). A mixed ordinal location scale model for analysis of Ecological Momentary Assessment (EMA) data. Statistics and Its Interface.

[CR25] Hedeker D, Gibbons RD (2006). Longitudinal data analysis.

[CR26] Hedeker D, Mermelstein RJ, Berbaum ML, Campbell RT (2009). Modeling mood variation associated with smoking: An application of a heterogeneous mixed-effects model for analysis of ecological momentary assessment (EMA) data. Addiction.

[CR27] Hedeker D, Mermelstein RJ, Demirtas H (2008). An application of a mixed-effects location scale model for analysis of ecological momentary assessment (EMA) data. Biometrics.

[CR28] Hedeker D, Nordgran R (2013). MIXREGLS: A program for mixed-effects location scale analysis. Journal of Statistical Software.

[CR29] Hofmann, R., & Nestler, S. (2019). Fluctuations in personality: The FLIP and the FLUX study (Tech. Rep.). Münster University of Münster.

[CR30] Hothorn T, Hornik K, Zeileis A (2006). Unbiased recursive partitioning: A conditional inference framework. Journal of Computational and Graphical Statistics.

[CR31] Hyndman RJ, Athanasopoulos G (2018). Forecasting: Principles and practice.

[CR32] Jahng S, Wood PK, Trull TJ (2008). Analysis of affective instability in ecological momentary assessment: Indices using successive difference and group comparison via multilevel modeling. Psychological Methods.

[CR33] James G, Witten D, Hastie T, Tibshirani R (2013). An introduction to statistical learning.

[CR34] Jiang J (2007). Linear and generalized linear mixed models and their applications.

[CR35] Kuhn M, Johnson K (2013). Applied predictive modeling.

[CR36] Leckie G, French R, Charlton C, Browne W (2014). Modeling heterogeneous variance-covariance components in two-level models. Journal of Educational and Behavioral Statistics.

[CR37] Li Y, Wang S, Song PX-K, Wanf N, Zhou L, Zhu J (2018). Doubly regularized estimation and selection in linear mixed-effects models for high-dimensional longitudinal data. Statistical Interface.

[CR38] McNeish DM (2015). Using LASSO for predictor selection and to assuage overfitting: A method long overlooked in behavioral sciences. Multivariate Behavioral Research.

[CR39] Nestler S (2020). Modeling interindividual differences in latent within-person variation: The confirmatory factor level variability model. British Journal of Mathematical and Statistical Psychology.

[CR40] Nestler S (2021). Modeling intraindividual variability in growth with measurement burst designs. Structural Equation Modeling.

[CR41] Ormerod JT, Wand MP (2010). Explaining variational approximations. The American Statistician.

[CR42] Pan J, Huang C (2014). Random effects selection in generalized linear mixed models via shrinkage penalty function Random effects selection in generalized linear mixed models via shrinkage penalty function. Statistical Computing.

[CR43] Pinheiro, J. , Bates, D. , DebRoy, S. , Sarkar, D., & R Core Team. (2020). nlme: Linear and nonlinear mixed effects models [Computer software manual]. https://CRAN.R-project.org/package=nlme R package version 3.1-148.

[CR44] Ram N, Gerstorf D (2009). Timestructured and net intraindividual variability: Tools for examining the development of dynamic characteristics and processes. Psychology and Aging.

[CR45] Scharf F, Nestler S (2019). Should regularization replace simple structure rotation in exploratory factor analysis?. Structural Equation Modeling: A Multidisciplinary Journal.

[CR46] Schelldorfer J, Bühlmann P, van de Geer S (2011). Estimation for high-dimensional linear mixed-effects models using L1-penalization. Scandinavian Journal of Statistics.

[CR47] Schelldorfer J, Meier L, Bühlmann P (2014). GLMMLasso: An algorithm for high-dimensional generalized linear mixed-effects models using L1-penalization. Journal of Computational and Graphical Statistics.

[CR48] Schölkopf B, Smola AJ (2002). Learning with Kernels: Support vector machines, regularization, optimization, and beyond.

[CR49] Schönbrodt FD, Gerstenberg FXR (2012). An IRT analysis of motive questionnaires: The unified motive scales. Journal of Research in Personality.

[CR50] Schuurman NK, Grasman RPPP, Hamaker EL (2016). A comparison of inverse-Wishart prior specifications for covariance matrices in multilevel autoregressive models. Multivariate Behavioral Research.

[CR51] Searle SR, Casella G, McCulloch CE (1992). Variance components.

[CR52] Sela, R. J., & Simonoff, J. S. (2011). REEMtree: Regression trees with random effects Computer software manual]. https://CRAN.R-project.org/package=REEMtree R package version 0.90.3.

[CR53] Sela RJ, Simonoff JS (2012). RE-EM trees: A data mining approach for longitudinal and clustered data. Machine learning.

[CR54] Skrondal A, Rabe-Hesketh S (2009). Prediction in multilevel generalized linear models. Journal of the Royal Statistical Society: Series A (Statistics in Society).

[CR55] Snijders TAB, Bosker RJ (2012). Multilevel analysis.

[CR56] Stegmann G, Jacobucci R, Serang S, Grimm KJ (2018). Recursive partitioning with nonlinear models of change. Multivariate Behavioral Research.

[CR57] Strobel C, Malley J, Tutz G (2009). An introduction to recursive partitioning: Rationale, application and characteristics of classification and regression trees, bagging and random forests. Psychological Methods.

[CR58] Therneau, T. & Atkinson, B. (2019). rpart: Recursive partitioning and regression trees [Computer software manual]. https://CRAN.R-project.org/package=rpart R package version 4.1-15.

[CR59] Tuerlinckx F, Rijmen F, Verbeke G, De Boeck P (2006). Statistical inference in generalized linear mixed models: A review. British Journal of Mathematical and Statistical Psychology.

[CR60] Vansteelandt K, Verbeke G (2016). A mixed model to disentangle variance and serial autocorrelation in affective instability using ecological momentary assessment data. Multivariate Behavioral Research.

[CR61] Verbeke G, Molenberghs G (2009). Linear mixed models for longitudinal data analysis.

[CR62] Wang LP, Bergeman CS, Hamaker E (2012). Investigating inter-individual differences in short-term intra-individual variability. Psychological Methods.

[CR63] Watson D, Clark LA, Tellegen A (1988). Development and validation of brief measures of positive and negative affect: The PANAS scales. Journal of Personality and Social Psychology.

